# Supplementary figures and images for: The impact of the newly developed school-based ‘Digital Health Contact’—Evaluating a health and wellbeing screening tool for adolescents in England
Source: PLoS One. 2024 Jan 12;19(1):e0297016. doi: 10.1371/journal.pone.0297016 (PMC10786370; doi:10.1371/journal.pone.0297016)

S1 Figure. DHC Flow Chart


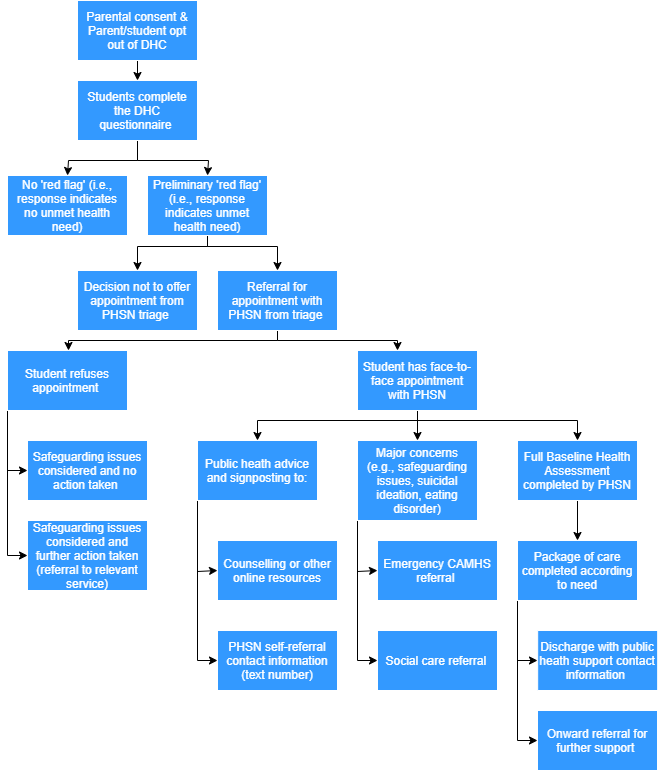

Supplement: S1 Fig — (DOCX) [file pone.0297016.s001.docx]
