# Supplementary material for: The impact of the newly developed school-based ‘Digital Health Contact’—Evaluating a health and wellbeing screening tool for adolescents in England
Source: PLoS One. 2024 Jan 12;19(1):e0297016. doi: 10.1371/journal.pone.0297016 (PMC10786370; doi:10.1371/journal.pone.0297016)
Supplement: S1 Table — School characteristics reported from 2018/19 government statistics. Little variation in school characteristics were observed across the three year data period. (DOCX) [file pone.0297016.s002.docx]

S1 Table. School-level characteristics

| **School** | **N times school took part in DHC** | **Years school took part in DHC** | **School size** | **% pupils eligible for free school meals** | **% pupils with English as first language** | **% girls** | **School IMD** | **% pupils with special educational needs** |
| --- | --- | --- | --- | --- | --- | --- | --- | --- |
| 1 | 3 | 2018/19, 2019/20, 2020/21 | 1319 | 12 | 92 | 46 | 8 | 9 |
| 2 | 2 | 2018/19, 2019/20 | 1399 | 13 | 10 | 47 | 4 | 12 |
| 3 | 2 | 2019/20, 2020/21 | 92 | 27 |  | 66 | 6 | 74 |
| 4 | 1 | 2019/20 | 1504 | 6 | 91 | 47 | 9 | 12 |
| 5 | 1 | 2019/20 | 958 | 11 | 98 | 49 | 10 | 13 |
| 6 | 1 | 2019/20 | 736 | 10 | 96 | 51 | 8 | 10 |
| 7 | 2 | 2018/19, 2020/21 | 1099 | 19 | 9 | 47 | 2 | 8 |
| 8 | 2 | 2018/19, 2019/21 | 842 | 19 | 46 | 9 | 4 | 7 |
| 9 | 1 | 2018/19 | 86 | 29 | 95 | 51 | 9 | 30 |
| 10 | 1 | 2019/20 | 1729 | 6 | 96 | 48 | 9 | 7 |
| 11 | 1 | 2018/19 | 590 | 15 | 97 | 48 | 8 | 16 |
| 12 | 1 | 2018/19 | 509 | 12 | 96 | 48 | 7 | 17 |
| 13 | 1 | 2018/19 | 587 | 21 | 83 | 44 | 5 | 20 |
| 14 | 1 | 2018/19 | 836 | 6 | 97 | 48 | 7 | 7 |
| 15 | 1 | 2018/19 | 962 | 6 | 97 | 48 | 3 | 7 |
| 16 | 1 | 2018/19 | 919 | 11 | 91 | 53 | 6 | 11 |
| 17 | 1 | 2018/19 | 816 | 10 | 99 | 51 | 7 | 14 |
| 18 | 1 | 2018/19 | 911 | 4 | 99 | 50 | 10 | 10 |
| 19 | 1 | 2018/19 | 645 | 14 | 94 | 53 | 6 | 13 |
| 20 | 1 | 2018/19 | 599 | 20 | 45 | 48 | 4 | 15 |
| 21 | 2 | 2018/19, 2020/21 | 960 | 28 | 66 | 52 | 1 | 14 |
| 22 | 0 | N/A | 1247 | 15 | 56 | 100 | 4 | 10 |
| 23 | 0 | N/A | 1390 | 10 | 60 | 44 | 7 | 9 |
| 24 | 0 | N/A | 1169 | 8 | 54 | 53 | 7 | 8 |
| 25 | 0 | N/A | 1790 | 13 | 28 | 47 | 4 | 11 |
| 26 | 0 | N/A | 1079 | 19 | 79 | 47 | 5 | 15 |
| 27 | 0 | N/A | 1504 | 16 | 24 | 49 | 3 | 11 |
| 28 | 0 | N/A | 1131 | 26 | 67 | 50 | 3 | 16 |
| 29 | 0 | N/A | 802 | 11 | 91 | 51 | 10 | 9 |
| 30 | 0 | N/A | 848 | 7 | 97 | 48 | 10 | 10 |
| 31 | 0 | N/A | 719 | 8 | 97 | 51 | 6 | 7 |
| 32 | 0 | N/A | 911 | 8 | 97 | 48 | 8 | 10 |
| 33 | 0 | N/A | 825 | 10 | 93 | 46 | 4 | 21 |
| 34 | 0 | N/A | 817 | 4 | 98 | 51 | 10 | 10 |
| 35 | 0 | N/A | 806 | 4 | 98 | 50 | 8 | 6 |
| 36 | 0 | N/A | 570 | 18 | 97 | 46 | 7 | 21 |

Abbreviations: DHC; Digital Health Contact

School characteristics reported from 2018/19 government statistics. Little variation in school characteristics were observed across the three year data period.
