# Supplementary material for: The impact of the newly developed school-based ‘Digital Health Contact’—Evaluating a health and wellbeing screening tool for adolescents in England
Source: PLoS One. 2024 Jan 12;19(1):e0297016. doi: 10.1371/journal.pone.0297016 (PMC10786370; doi:10.1371/journal.pone.0297016)
Supplement: S2 Table — Five separate doubly robust interaction models were conducted due to the limited sample size. Only interaction effects are presented. Main effects are not presented. All models were adjusted for year, number of pupils taking part in the DHC, ever taken part in DHC, % pupils eligible for FSM, % pupils with English as first language, % girls, % pupils with SEN and school IMD. (DOCX) [file pone.0297016.s003.docx]

S2 Table. Doubly robust negative binomial mixed-effects regression models exploring interactions between taking part in the DHC and school-level covariates on the annual number of PHN(SN) referrals (n observations = 102, n schools = 35)

| **Number of annual PHN(SN) referrals** | **Coefficient** | **95 % CI** | | **p value** |
| --- | --- | --- | --- | --- |
| Model 1: DHC x % pupils eligible for FSM |  |  |  |  |
| Taking part in DHC - high | -0.08 | -0.53 | 0.37 | 0.732 |
| Model 2: DHC x % pupils with English as first language |  |  |  |  |
| Taking part in DHC - high | -0.55 | -1.02 | -0.09 | 0.020 |
| Model 3: DHC x % girls |  |  |  |  |
| Taking part in DHC - high | -0.39 | -1.04 | 0.26 | 0.238 |
| Model 4: DHC x School IMD |  |  |  |  |
| Taking part in DHC - high | -0.40 | -0.88 | 0.08 | 0.103 |
| Model 5: DHC x % pupils with SEN |  |  |  |  |
| Taking part in DHC - high | -0.39 | -0.97 | 0.19 | 0.185 |

Abbreviations: DHC; Digital Health Contact, PHN(SN); Public Health Nurse (School Nursing)

Interaction terms were added individually to doubly robust models due to the limited sample size. Only interaction effects are presented. Main effects are not presented. All models were adjusted for year, number of pupils taking part in the DHC, ever taken part in DHC, % pupils eligible for FSM, % pupils with English as first language, % girls, % pupils with SEN and school IMD.
