# Supplementary material for: The impact of the newly developed school-based ‘Digital Health Contact’—Evaluating a health and wellbeing screening tool for adolescents in England
Source: PLoS One. 2024 Jan 12;19(1):e0297016. doi: 10.1371/journal.pone.0297016 (PMC10786370; doi:10.1371/journal.pone.0297016)
Supplement: S3 Table — *Green = N decreased (improved), Blue = N stayed the same, Red = N increased (worsened). (DOCX) [file pone.0297016.s004.docx]

**S3 Table. Change in number of red flags between Year 9 and Year 11 among the sample of 164 pupils**

| **Number of pupils with red flags in Year 9** | **Number of pupils with red flags in Year 11** | | | | | | | | |
| --- | --- | --- | --- | --- | --- | --- | --- | --- | --- |
|  | **0 red flags** | **1 red flag** | **2 red flags** | **3 red flags** | **4 red flags** | **5 red flags** | **6 red flags** | **7 red flags** | **Total** |
| 0 red flags | 67 | 8 | 7 | 2 | 0 | 1 | 0 | 0 | 85 |
| 1 red flag | 14 | 4 | 5 | 3 | 2 | 2 | 1 | 0 | 31 |
| 2 red flags | 8 | 3 | 4 | 0 | 2 | 0 | 0 | 0 | 17 |
| 3 red flags | 2 | 2 | 2 | 4 | 4 | 0 | 0 | 0 | 14 |
| 4 red flags | 1 | 5 | 2 | 0 | 2 | 0 | 1 | 1 | 12 |
| 5 red flags | 0 | 0 | 1 | 2 | 1 | 0 | 0 | 0 | 4 |
| 6 red flags | 0 | 0 | 0 | 0 | 0 | 0 | 0 | 1 | 1 |
| Total | 92 | 22 | 21 | 11 | 11 | 3 | 2 | 2 | 164 |

*Green = N decreased (improv­­­ed), Blue = N stayed the same, Red = N increased (worsened)
