# Supplementary material for: The impact of the newly developed school-based ‘Digital Health Contact’—Evaluating a health and wellbeing screening tool for adolescents in England
Source: PLoS One. 2024 Jan 12;19(1):e0297016. doi: 10.1371/journal.pone.0297016 (PMC10786370; doi:10.1371/journal.pone.0297016)
Supplement: S4 Table — (DOCX) [file pone.0297016.s005.docx]

S4 Table. Principle components, showing correlations between DHC items, completed by pupils in Year 9 and again in Year 11 (n=164)

|  | **Year 9** | | **Year 11** | |
| --- | --- | --- | --- | --- |
| **DHC questions that can result in a red flag** | **Principle component 1** | **Principle component 2** | **Principle component 3** | **Principle component 4** |
| Feeling safe at home | -0.31 |  |  |  |
| Feeling safe at school | -0.39 |  |  |  |
| Feeling safe online |  | 0.44 |  |  |
| Being bullied at school |  | 0.41 |  |  |
| In a sexual relationship |  |  |  |  |
| Worried about child sexual exploitation |  |  |  |  |
| Regularly feel worries/anxious | 0.37 |  | 0.46 |  |
| Regularly in low mood | 0.43 |  | 0.48 |  |
| Worries about body image | 0.34 | -0.37 | 0.41 |  |
| Worries about puberty |  | 0.45 |  |  |
| Worries about sexuality |  | -0.33 |  | 0.54 |
| Self-harm | 0.31 |  | 0.38 |  |
| Worried about disabilities affecting school |  |  |  | 0.66 |

Abbreviations: DHC; Digital Health Contact
