# Supplementary material for: The impact of the newly developed school-based ‘Digital Health Contact’—Evaluating a health and wellbeing screening tool for adolescents in England
Source: PLoS One. 2024 Jan 12;19(1):e0297016. doi: 10.1371/journal.pone.0297016 (PMC10786370; doi:10.1371/journal.pone.0297016)
Supplement: Attachment — Submitted filename: Response to Reviewers.docx [file pone.0297016.s006.docx]

**Response to Reviewers**

**PONE-D-23-02654**

**The impact of the newly developed school-based ‘Digital Health Contact’ – evaluating a health and wellbeing screening tool for adolescents in England**

**PLOS ONE**

We would like to thank the editor and reviewers for taking their time to review our manuscript. We have addressed all the suggestions made and highlight where these changes can be found in the manuscript/supporting documents. We hope you feel we have addressed your suggestions fully and that our manuscript will soon by published in PLOS ONE. Thank you.

**Comments from Editor:**

1. Please ensure that your manuscript meets PLOS ONE's style requirements, including those for file naming. The PLOS ONE style templates can be found at

<https://journals.plos.org/plosone/s/file?id=wjVg/PLOSOne_formatting_sample_main_body.pdf> and

<https://journals.plos.org/plosone/s/file?id=ba62/PLOSOne_formatting_sample_title_authors_affiliations.pdf>.

**Response:**

We apologise that our manuscript did not meet PLOS ONE’s style requirements. We have now reformatted the manuscript in accordance with the style requirements. We updated the title page, changed the level 1 and level 2 headings, changed ‘Figure’ to ‘Fig’, changed the Supplementary Figures and Tables to ‘S1 Fig’, ‘S1 Table’ etc and uploaded the Figure as a TIFF file.

2. Thank you for providing the following Funding Statement:

“AP, KD, PA, NW, HF, KB and FDV have declared that no competing interests exists.
I have read the journal's policy and authors CM and ST have the following competing interests: CM is employed at the DHCs commissioning organisation (at Leicester City Council), and ST is employed at the DHCs provider organisation (at Leicestershire Partnership NHS Trust). This study, and the wider research project evaluating the DHC, has been co-produced with the DHC’s commissioners (at Leicester City Council) and providers (at Leicestershire Partnership NHS Trust). The commissioner and the provider are committed to helping to facilitate a robust evaluation of the DHC in order to inform potential changes and wider roll out of the programme. There has been no pressure or influence to modify, restate, weaken, omit or frame findings, conclusions and recommendations from any team member.”

We note that one or more of the authors is affiliated with the funding organization, indicating the funder may have had some role in the design, data collection, analysis or preparation of your manuscript for publication; in other words, the funder played an indirect role through the participation of the co-authors.

If the funding organization did not play a role in the study design, data collection and analysis, decision to publish, or preparation of the manuscript and only provided financial support in the form of authors' salaries and/or research materials, please review your statements relating to the author contributions, and ensure you have specifically and accurately indicated the role(s) that these authors had in your study in the Author Contributions section of the online submission form. Please make any necessary amendments directly within this section of the online submission form.  Please also update your Funding Statement to include the following statement: “The funder provided support in the form of salaries for authors [insert relevant initials], but did not have any additional role in the study design, data collection and analysis, decision to publish, or preparation of the manuscript. The specific roles of these authors are articulated in the ‘author contributions’ section.”

If the funding organization did have an additional role, please state and explain that role within your Funding Statement.

Please also provide an updated Competing Interests Statement declaring this commercial affiliation along with any other relevant declarations relating to employment, consultancy, patents, products in development, or marketed products, etc.

Within your Competing Interests Statement, please confirm that this commercial affiliation does not alter your adherence to all PLOS ONE policies on sharing data and materials by including the following statement: "This does not alter our adherence to  PLOS ONE policies on sharing data and materials.” (as detailed online in our guide for authors <http://journals.plos.org/plosone/s/competing-interests>). If this adherence statement is not accurate and  there are restrictions on sharing of data and/or materials, please state these. Please note that we cannot proceed with consideration of your article until this information has been declared.

**Response:**

Apologies for the slight confusion between our funding statement and conflict of interest statement. Our funding statement was submitted as “This project is funded by the National Institute for Health Research (NIHR) School for Public Health Research (SPHR) (SPHR-PHPES009-DHC).”

The conflicts of interest statement is separate to the funding statement and read “AP, KD, PA, NW, HF, KB and FDV have no competing interests. CM is employed at the DHCs commissioning organisation (at Leicester City Council), and ST is employed at the DHCs provider organisation (at Leicestershire Partnership NHS Trust). This study, and the wider research project evaluating the DHC, has been co-produced with the DHC’s commissioners (at Leicester City Council) and providers (at Leicestershire Partnership NHS Trust). The commissioner and the provider are committed to helping to facilitate a robust evaluation of the DHC in order to inform potential changes and wider roll out of the programme. There has been no pressure or influence to modify, restate, weaken, omit or frame findings, conclusions and recommendations from any team member.”

Please note that the funders of this project (NIHR SPHR) had no role in the project. In our conflicts of interest statement we highlight that CM and ST were associated with the service/intervention (i.e. the Digital Health Contact) that we were evaluating and that they provided impartial input into the co-production of the evaluation. CM and ST are not associated with the funders. This study was not funded by Leicester City Council or Leicestershire Partnership NHS Trust.

In the Revised Info for submission, we have updated our funding statement to: “This study is funded by the National Institute for Health and Care Research (NIHR) School for Public Health Research (SPHR) (Grant Reference Number SPHR-PHPES009-DHC). The grant was awarded to FDV. The NIHR School for Public Health Research is a partnership between the Universities of Sheffield, Bristol, Cambridge, Imperial, University College London, The London School for Hygiene and Tropical Medicine (LSHTM), LiLaC — a collaboration between the Universities of Liverpool and Lancaster and The Centre for Translational Research in Public Health (Fuse) a collaboration between Newcastle, Durham, Northumbria, Sunderland and Teesside Universities. The views expressed are those of the author(s) and not necessarily those of the NIHR or the Department of Health and Social Care. The funders had no role in study design, data collection and analysis, decision to publish, or preparation of the manuscript.”

And edited our conflict of interest statement to: “AP, KD, PA, NW, HF, KB and FDV have no competing interests. The funders had no additional role in this study. We should note that CM is employed at the DHCs commissioning organisation (at Leicester City Council), and ST is employed at the DHCs provider organisation (at Leicestershire Partnership NHS Trust). This study, and the wider research project evaluating the DHC, has been co-produced with the DHC’s commissioners (at Leicester City Council) and providers (at Leicestershire Partnership NHS Trust). The commissioner and the provider are committed to helping to facilitate a robust evaluation of the DHC in order to inform potential changes and wider roll out of the programme. There has been no pressure or influence to modify, restate, weaken, omit or frame findings, conclusions and recommendations from any team member. The study did not receive funding from the commissioner or provider.”

**Comments from Reviewer 1 and our responses**

Dear Reviewer 1, thank you for taking time to review our manuscript. We have responded to your specific comments in the table below.

| **Reviewer 1 Comments** | **Our Responses** |
| --- | --- |
| Abstract: - The manuscript does not have an abstract, making it difficult for the reader to understand what your manuscript will discuss. | Please find the abstract on pages 1-2 of the revised manuscript document (lines 17-42) |
| Introduction: - It’s too short and does not fully explain what your paper will discuss.  - I suggest you change the title of the introduction to Abstract, then after finishing the Abstract part (After discussion). You should write an introduction describing what your paper will discuss. | Please find a full Introduction on pages 3-5 under the heading Introduction (lines 42-100)  The Introduction appears in the revised manuscript document after the Abstract |
| Background: - The structure of the paper is not numbered and is very confusing. | We have now changed this heading to Introduction, which refers to the section you are referring to in the above comment. This section is not required to be numbered as it provides background literature to the study and then describes the study aims. |
| Methods: - It should contain an introductory paragraph to illustrate what this section will discuss. | We have included an introductory paragraph on page 5 (lines 101-102), which reads “We describe the methods of a real-world evaluation study, including the study design, recruitment strategy, measures and statistical analysis.” |
| Results - It should contain an introductory paragraph to illustrate what this section will discuss. | We have included a introductory sentence on page 8 (line 176), which reads “We presents the results of the school-level, pupil-level, and principle component analyses.” |
| Conclusion: The future development of your work should be clarified more. Moreover, some achieved results should be mentioned in this part. | We have added two additional sentences to the Conclusion on pages 19-20 (lines 345-355). The first sentence at the start of the paragraph reads “Our findings suggest that the DHC did not impact referrals to the Public Health School Nurses and did not reduce the number of times pupils were raised as ‘red flags’ on the DHC survey from Year 9 to Year 11.”. The second sentence at the end of the paragraph reads “Further co-production work with schools and pupils to improve the implementation and effectiveness of the DHC should be conducted before further roll-out of the programme to other cities in the UK.” |

**Comments from Reviewer 2 and our responses**

Dear Reviewer 2, thank you for your comment, we are glad you found the manuscript to be well written and clearly described.
